# Geochemical Mass Balance and Elemental Transport during the Weathering of the Black Shale of Shuijingtuo Formation in Northeast Chongqing, China

**DOI:** 10.1155/2014/742950

**Published:** 2014-08-13

**Authors:** Sixiang Ling, Xiyong Wu, Siyuan Zhao, Xin Liao, Yong Ren, Baolong Zhu

**Affiliations:** ^1^Department of Geological Engineering, Southwest Jiaotong University, Chengdu 611756, China; ^2^Moe Key Laboratory of High-Speed Railway Engineering, Southwest Jiaotong University, Chengdu 610031, China; ^3^School of Civil Engineering and Architecture, Southwest University of Science and Technology, Mianyang 621010, China

## Abstract

An understanding of the processes that control the behavior of major elements with respect to weathering profile is essential to calculate the mobility, redistribution, and mass fluxes of elements. Hence, this study aims to determine the geochemical mass balance, strain, elemental correlation, and transport in weathering profiles. We constructed three weathering profiles for the black shale of Shujingtuo formation. As per the principal component analysis of major elements, density, and pH values, the first component represents the “elemental factor” and the second denotes the “external factor.” The “depletion” pattern is a mass transportation pattern, and Na, K, and Mg are depleted along transect relative to the composition of fresh rock. Fe is redeposited at the bottom half of the saprock zone, whereas Al is accumulated at the regolith zone. The Fe and Al patterns are attributed to the “depletion–addition” and “addition” patterns, respectively. The strain in profiles A and B demonstrates the expansion at the regolith zone and part of the saprock zone. In profile C, however, these zones collapsed at all depths. In chemical weathering, Na, K, Ca, Mg, and Si are depleted in the following order: valley (C) > near mountaintop (B) > ridge (A).

## 1. Introduction

The chemical weathering of rocks is a major process that alters the Earth's surface and is a critical process in the geochemical cycling of elements [1]. It is also the main geochemical process in the weathering of black shale. Black shale is a common sedimentary rock on the Earth's surface that contains organic matter, pyrite, and other sulfide minerals [2, 3]. In the oxygen-rich supergene environment of black shale, sulfide minerals oxidize and produce sulfuric acid fluids with a low pH value. These fluids dissolve rock-forming minerals and deteriorate rock through the hydrolysis of clays and silicate and the concomitant leaching of alkali and alkaline earth elements [4–6]. Accompanied by the complexity of chemical weathering interactions, certain element groups display similar patterns because of their identical mineralogical origins, common chemical processes, and similar geochemical properties. Based on elemental migration and mineralogical transformation, these patterns have been classified as “immobile,” “depletion,” “addition,” “depletion—addition,” and “biogenic” profiles [7]. Thus, many field and laboratory studies have investigated elemental mobility in sedimentary rocks during chemical weathering under neutral to acidic conditions, including black shale [8–11].

Black shale is widely distributed in Southern China [12, 13], and its chemical weathering has been aggravated by the heavy rainfall and humid weather conditions along with element leaching [14]. In this study, we construct three weathering profiles of black shale in Chengkou County, Northeast Chongqing Province, China. To identify the geochemical behaviors of different major element groups during black shale weathering, the concentrations of major elements were determined in fresh rock, weathered shale, saprock, and regolith from the study area. This study enhances understanding regarding elemental mobilization, redistribution, strain behaviors, and mass gain or loss fluxes during the chemical weathering of black shale.

## 2. Field Site

**Figure 1:**
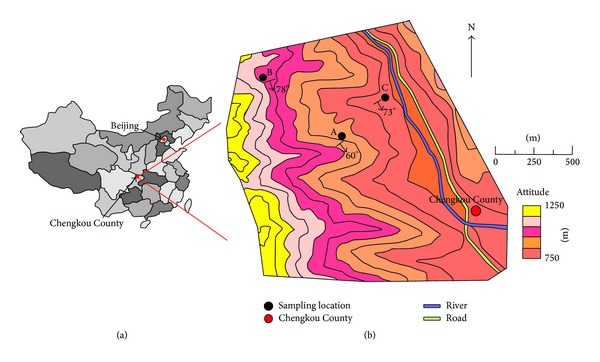
Study site on Shuijingtuo Formation black shale in Chengkou county, Northeast Chongqing Province. The samples were collected from nearby mountaintop (B), midridge (A), and valley floor (C). Background color indicates altitude.

The study was conducted in Chengkou County of Northeast Chongqing Province, which is located at the boundary between the Qinling Orogenic Belt and the Yangtze Platform. The site is characterized by rugged topography and elevations that are 750 m–1200 m above sea level. The latitude is 31°57′N-31°58′N, and the longitude is 108°37′E–108°39′E. Specifically, profiles A, B, and C are located at 31°57′27′′N and 108°38′10′′E (ridge), 31°57′46′′N and 108°37′42′′E (nearby mountaintop), and 31°57′39′′N and 108°38′39′′E (valley), respectively (Figure 1). The catchment experiences a subtropical mountain monsoon climate with a mean annual air temperature of 13.8°C and average annual rainfall of 1261.4 mm. The warm and moist climate facilitates the chemical weathering of black shale. The catchment overlies black shale from the Shuijingtuo Formation of the Qiongzhusi Group in Lower Cambrian. The slope gradient generally varies from 20° to 45°, and the average local relief from the valley floor to the ridge top is approximately 500 m. The study field is currently covered with a mixed broad-leaved/shrub forest. Furthermore, the Renhe River flows over the county from the south to the northwest and eventually merges with the Yangtze River via the Hanjiang.

## 3. Sample Preparation and Analysis Methods

The profile material can be divided into regolith, saprock, weathered shale, and fresh shale (protolith) according to the field investigation. The regolith portions of profiles A, B, and C range from 0 m to 0.25 m, 0 m to 2 m, and 0 m to 1.5 m, respectively. The saprock portions in these profiles range from 0.25 m to 1.5 m, 2 m to 4.8 m, and 1.5 m to 6.2 m. The weathered shale portions range from 1.5 m to 3 m, 4.8 m to 7 m, and 6.2 m to 10.5 m. The fresh shale portions are below 3, 7, and 10.5 m. Samples were collected from these material zones for three profiles, and the sample number and depth are shown in Table 1. After collection, the solid samples were dried in air and ground to pass through a 100-mesh sieve (<150 *μ*m). The pH values of the shale were measured in a slurry of shale powder and deionized water (1 : 2.5) (NY/T 1377–2007) by a HACH HQ30d pH meter [15]. The weathered rock and protolith mineralogy were qualitatively characterized by powder X-ray diffraction (XRD) in oriented samples on a Rigaku D/MAX-2500 X-ray with a Cu filament and Ni filter. To analyze the elemental chemistry of weathered rock and the parent shale, the ground powders were fused with lithium borate flux (50%–50% Li_2_B_4_O_7_–LiBO_2_) in an autofluxer at temperatures ranging between 1050°C and 1100°C. A flat molten glass disc was prepared from the resulting melt and analyzed for elemental concentrations through X-ray fluorescence spectrometry (XRF) on a PANalytical Axios Max apparatus at ALS Minerals-ALS Chemex (Guangzhou) Co. Ltd. Reference rocks with known compositions (SARM-3, SARM-32, and SARM-45) were processed using the same protocol and were utilized as calibration standards. The detection limit is approximately 0.01%, and the precision of all XRF analyses was <0.5% except in relation to SO_3_ and Fe_2_O_3_ (<3.6%). Ti was detected by inductively coupled plasma with mass spectrometry (ICP-MS).

The samples were collected from the field at various depths to measure bulk density through the wax-sealing method. The samples were weighed after drying at 105°C in a drying oven for 24 h. They were then covered with wax and weighed in air and water. Given the known density of water and wax, the rock volumes were calculated according to the difference between the volumes of rocks covered with wax and the volumes of wax.

## 4. Results and Discussion

### 4.1. Elemental Characteristics in the Profile

The major element concentrations, including the mean chemical composition of the upper continental crust (UCC), are listed in Table 1. As per the XRD experiment, the primary minerals in profiles A, B, and C are quartz, albite, muscovite, calcite, dolomite, and pyrite, whereas the secondary minerals include gypsum, goethite, illite, and smectite. In profile C, the weathered shale and protolith portions contained fluorapatite. Based on the XRF analysis (Table 1), Si content increases from 52.3% to 65.2% in profile A and from 49.1% to 70.5% in profile C. However, it remains relatively stable in profile B (66.0%–69.7%). Al_2_O_3_ concentrations increased gradually from protolith to regolith at a range of 4.51%–17.5%. Fe_2_O_3_ concentrations in profiles A, B, and C ranged from 1.35% to 12.06%, 2.87% to 5.94%, and 0.87% to 9.91%, respectively. Na_2_O concentrations ranged from 1.94% to 2.94%, 0.42% to 2.29%, and 0.09% to 0.83%. K_2_O concentration is similar to that of Na_2_O and is depleted from fresh shale to regolith. Ca, which is principally bound to calcite in the protolith and to gypsum in the secondary phase, is depleted to concentrations of less than 0.29% in the regolith zone of profile A. Furthermore, its concentration was less than 0.41% in profile C, except on the surface sample. MgO is similarly depleted from the protolith to the regolith zone.

**Figure 2:**
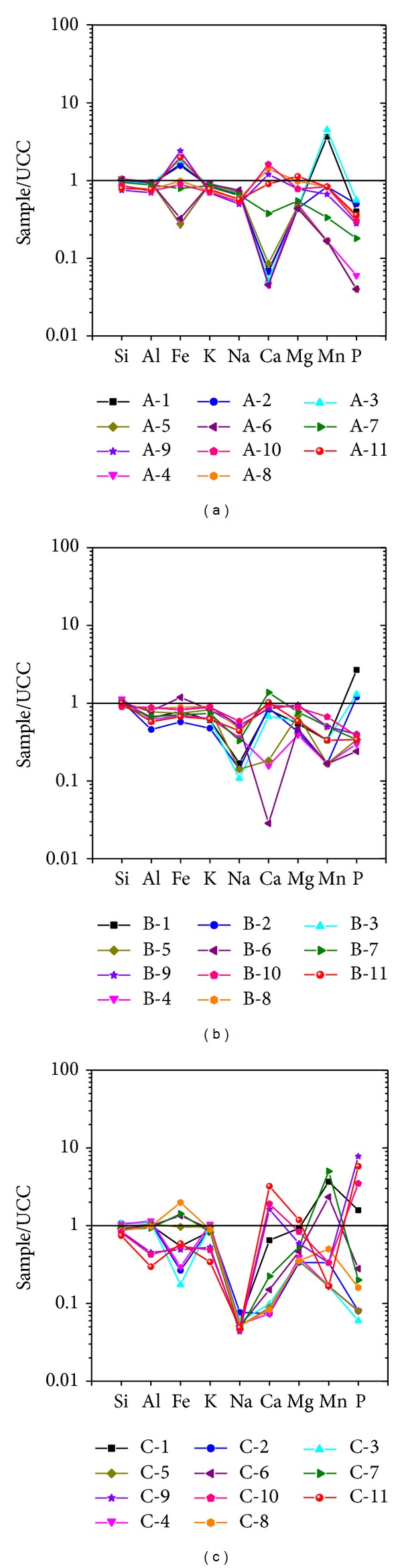
Spider plots showing the UCC-normalized pattern of major elements for each sample of the Shuijintuo Formation black shale in Chengkou County.

The ratio of element contents to UCC can explain the variation in the weathering of protolith and of weathered rock [16, 17]. The results of the UCC-normalized patterns of major elements are shown in Figure 2. The concentrations of Si, Al, and K are close to those in the chemical composition of the UCC in profile A (Figure 2(a)). Fe concentration in the protolith is greater than that in the chemical composition of the UCC, but it gradually decreases with decreasing depth. The concentrations of the alkaline earth elements Mg and Ca in the protolith are close to those in the chemical composition of the UCC. In weathered rock, the concentrations of these elements are almost less than those in the chemical composition of the UCC. The concentrations of Na and P are less than those in the chemical composition of the UCC. The concentration of Mn in protolith is close to that in the chemical composition of the UCC. From A-10 to A-4 in the weathered shale zone and the bottom half of the saprock zone of profile A, this concentration was less than that in the chemical composition of the UCC. However, it exceeded that in the chemical composition of the UCC from A-3 to A-1, which belongs to the regolith zone and the upper half of the saprock zone (Figure 2(a)). In profile B, the concentrations of Si, Al, and Fe are close to those in the chemical composition of the UCC (Figure 2(b)). The concentrations of K, Na, Ca, Mg, and Mn are almost less than those in the chemical composition of the UCC. The concentration of P in protolith and in some weathered rocks is less than that in the chemical composition of the UCC. However, it is considerably greater than that in the chemical composition of the UCC in the regolith zone (Figure 2(b)). In profile C, the concentration of Si is close to that in the chemical composition of the UCC (Figure 2(c)). The concentrations of Al, Fe, K, Na, and Mn in protolith (C-11) are less than those in the chemical composition of the UCC, whereas the concentrations of Fe and Mn are greater than those in the chemical composition of the UCC at the saprock zone. The concentrations of Ca, Mg, and P in protolith are greater than those in the chemical composition of the UCC, but they decrease with decreasing depth (Figure 2(c)). However, Al accumulated gradually during the weathering process. In profile C, however, the concentration of P in protolith is greater than that in the chemical composition of the UCC because the fluorapatite mineral is retained in the shale during the XRD experiment. During geological weathering in the study area, the concentrations of Si and Al are relatively stable in profiles A, B, and C. Nonetheless, the UCC may lose Na and K during redeposition and diagenesis in an anoxic environment. The concentrations of Fe, Mn, and P deviated significantly from those in the chemical composition of the UCC, thus demonstrating the effect of continental chemical weathering. In addition, the concentrations of Mn and P are greater than those in the chemical composition of the UCC near the surface zone because of environment pollution and the enrichment of fresh organic matter during chemical weathering.

### 4.2. Element Migration along Profiles

Element mobility in the catchment is characterized by the mass transfer coefficient *τ*_*i*,*j*,_ which is computed based on chemical composition data. The migrative elements result in the residual enhancement of comparatively stable elements, and *τ*_*i*,*j*_ may distort the real characteristic of element migration or enrichment [16]. Hence, the value of *τ*_*i*,*j*_ represents elemental enrichment or depletion throughout the profile relative to elemental concentration in the fresh rock or protolith material. The value of *τ*_*i*,*j*_ is calculated as follows [18–20]:

(1) τi,j=Cj,wCi,pCj,pCi,w−1,

where *τ*_*i*,*j*_ is a dimensionless coefficient that represents the concentration ratio and *C* refers to concentrations of either mobile or immobile elements (*j* and *i*, resp.) in either weathered samples (subscript *w*) or protolith (subscript *p*). We assume that Ti is the immobile element (*i*), as Ti was mainly inert during weathering. Positive *τ*_*i*,*j*_ values denote the enrichment of element (*j*) in the transported and residual material of the “open-system,” whereas negative values indicate depletion in the profile [16, 21]. When *τ*_*i*,*j*_ is −1.0, all of the original mass of element *j* is completely depleted in the protolith during weathering. A value of zero suggests that element *j* is neither enriched nor depleted in protolith.

**Figure 3:**
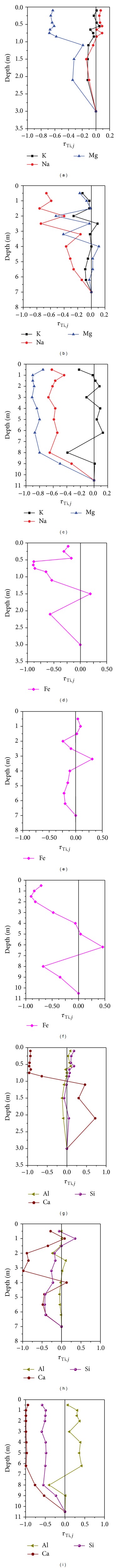
Depth profiles for Tau values for major elements in profile A (left panel: (a), (d), and (g)), B (middle panel: (b), (e), and (h)), and C (right panel: (c), (f), and (i)). A = ridge; B = near mountaintop; C = valley.

The Tau values of corresponding major elements are listed in Table 1 and shown in Figure 3. The results show that *τ*_*i*,*j*_ < 0 in most elements, which indicates that these elements were depleted during chemical weathering (Figure 3). The *τ*_Ti,*j*_ value of the K element varies between −0.39 and 0.13, which suggests that K was either leached slightly or was quite stable during weathering. The *τ*_Ti,*j*_ values of Na and Mg ranges between −0.76 and 0.09 and between −0.9 and 0.11, respectively. This result indicates that Na and Mg are strongly depleted during weathering. Therefore, K, Na, and Mg can be classified under “depletion” profiles in the study area (Figures 3(a), 3(b), and 3(c)) [7]. The *τ*_Ti,*j*_ value of Fe is greater than 0 in the saprock zone but less than 0 in the other zones of all three profiles, with the exception of the regolith zone in profile B because pyrite dissolved in the regolith zone. Furthermore, Fe-oxyhydroxide, or goethite minerals are produced and redeposited in the saprock zone, and mafic silicates may disintegrate; thus, Fe(*Ι* *Ι*) is released and transferred downwards, rapidly oxidized, and either precipitated as hydrous oxide or adsorbed by clay minerals in the saprock zone [10]. Consequently, the mass transportation pattern of Fe can be considered a “depletion-addition” pattern (Figures 3(d), 3(e), and 3(f)). The maximum *τ*_Ti,*j*_ values of Ca are 0.72 and 0.13 in the saprock and weathered shale zones of profiles A and B. However, the *τ*_Ti,*j*_ value of Ca is less than 0 at other depths because the calcite mineral dissolves in the regolith zone and gypsum is formed at the saprock and weathered shale zones (Table 1). Therefore, the *τ*_Ti,*j*_ pattern for Ca in these profiles can be regarded as a “depletion-addition” pattern (Figures 3(g) and 3(h)). However, the *τ*_Ti,*j*_ value of Ca is less than 0 in profile C; therefore, it is classified under the “depletion” pattern (Figure 3(i)). Given Al, the *τ*_Ti,*j*_ values range from −0.12 to 0.10 and from −0.23 to 0.11 in profiles A and B, respectively, thus indicating little to no loss or gain in this element. In profile C, however, Al accumulates in the regolith, saprock, and weathered shale zones. This finding suggests that the pattern of Al is an “addition” pattern (Figure 3(i)). The *τ*_Ti,*j*_ of Si is expressed as *τ*_A_≈−0.07–0.18 > *τ*_B_≈ −0.43–0 >*τ*_C_ ≈ −0.59–−0.23 in the three profiles, thereby indicating that the quartz mineral remained unchanged in profile A but it was strongly depleted in profiles B and C. As per the *τ*_Ti,*j*_ values of all of the elements, the elements of alkali and alkaline earth are highly depleted in all profiles. In particular, Ca and Mg were almost completely depleted. Moreover, |*τ*_Ti,*j*_ | >0.2 in Ca, Mg, and Na, thus suggesting that these elements migrate actively and are highly active migration elements. |*τ*_Ti,*j*_ | <0.1 is almost true for K, which demonstrates that this element migrated slightly as a moderately active migration element [16]. Based on the *τ*_Ti,*j*_ values of each element, the activity and migration sequence of each element during chemical weathering are as follows: Ca ≈ Mg > Na > K > Si > Al.

### 4.3. Principal Component Analysis (PCA)

PCA is a conventional multivariate technique that is applied to study geochemical data [22, 23]. PCA cuts down a large number of variables to determine the components (groups of variables) that influence variations in multivariate data [24]. PCA is founded on the correlation matrix, which measures interrelationships among multiple variables. The first principal component explains most of the variance within original data, and each subsequent principal component describes the variance in progressively limited detail. Multivariate data can usually be simplified into two or three principal components that account for the majority of the variance in data.

We examined chemical composition, density, and pH data in each individual sampling medium through Pearson correlation analysis (Table 2). In profile A, SiO_2_, and Al_2_O_3_ are strongly and positively correlated (*r* = 1.0~0.800) with alkali elements but are strongly and negatively correlated with alkaline earth elements, density, and pH value. The SiO_2_ and Al_2_O_3_ in profile C display similar correlations with major oxides and density. In profile B, SiO_2_ is negatively correlated with all major oxides, density, and pH value, with the exception of Al_2_O_3_ and P_2_O_5_. Al_2_O_3_ is positively correlated with alkali elements and density and is moderately and positively correlated (*r* = 0.799~0.600) with Fe_2_O_3_ (Table 2). However, it is negatively correlated with alkaline earth elements and pH value. In all three profiles, SO_3_ and loss on ignition (LOI) are positively correlated with alkaline earth elements, Fe_2_O_3_, and density but are negatively correlated with alkali elements, with the exception of profile B. The Fe_2_O_3_–pH correlation coefficients are −0.569, −0.764, and −0.728 in profiles A, B, and C, respectively. Therefore, Fe_2_O_3_ is negatively correlated with pH value and positively correlated with SO_3_ but is either negatively or positively correlated with other oxides in all three profiles. The silicate mineral elements are negatively correlated with pH value. Nonetheless, the alkaline earth elements are positively correlated with this value.

Subsequently, the average values of the different sampling media were examined through PCA. The results reported in Table 4 show that PC1 and PC2 account for 72.84% and 17.88%, respectively, of the total variance in profile A. In profile B, PC1 and PC2 explain 48.22% and 25.52%, respectively, of the total variance in the data. In profile C, PC1, and PC2 correspond to 63.76% and 25.61%, respectively, of total variance. The Si concentrations in PC1 are −0.967, −0.892, and −0.921 in profiles A, B, and C, respectively. Moreover, the concentration of alkaline earth elements, density, and the LOI of PC1 exceed 0.8, 0.588, and 0.322, respectively. Consequently, the amount of Si loaded on PC1 is negative. By contrast, the loadings of Ca, Mg, SO_3_, LOI, density, and pH components are positive on the PC1 of data in individual sampling media (Table 3). The concentrations of the alkali elements of PC1 are greater than 0.833 in profile B and are less than −0.618 in profiles A and C. Therefore, the loading of alkali elements is negative on the PC1 of data in profiles A and C, whereas it is positive on the PC1 in profile B. The concentrations of Al in PC1 are −0.989, 0.862, and −0.983 in profiles A, B, and C, respectively. The PC1 of the individual datasets reveals that Na, K, Mg, and Ca are unstable, whereas Si and Al are stable. PC1 represents the “elemental factor” that affects the mobility of the chemical properties of elements during chemical weathering. Fe and pH value are most significantly loaded on the PC2 of data associated with individual sampling media. The Fe concentrations in PC2 are −0.771, −0.716, and −0.942 in profiles A, B, and C, respectively. The loading of the pH value of PC2 is most significant and positive (0.899) with respect to chemical weathering. The loading of the density of PC2 in the profiles A and B is positive but slightly negative in profile C. Therefore, PC2 is influenced by environmental changes. PC2 represents the “external factor” that may be related to the mobility of major elements under varying conditions of chemical alteration, that is, residual enrichment, pH, temperature, Eh, ion complexes, and so forth. In addition, the low pH enhances element variation and accelerates chemical weathering. PC3 and PC4 denote either physical or biological weathering, as well as other factors. The PCA results show that pH value and alkali and alkaline earth elements significantly influence chemical weathering and that Mg, Ca, Na, and K are almost depleted or depleted-added in the weathering process.

### 4.4. Strain Factor

During chemical weathering, the changes in volume from the protolith (*V*_*p*_) to the weathered sample (*V*_*w*_) can be assessed by calculating the strain factor *ε*_*i*,*w*_ [11, 21]. Therefore, the volumetric strain can be derived from the ratios of densities and concentrations of inert element *i* in the weathered rocks and protolith to obtain

**Figure 4:**
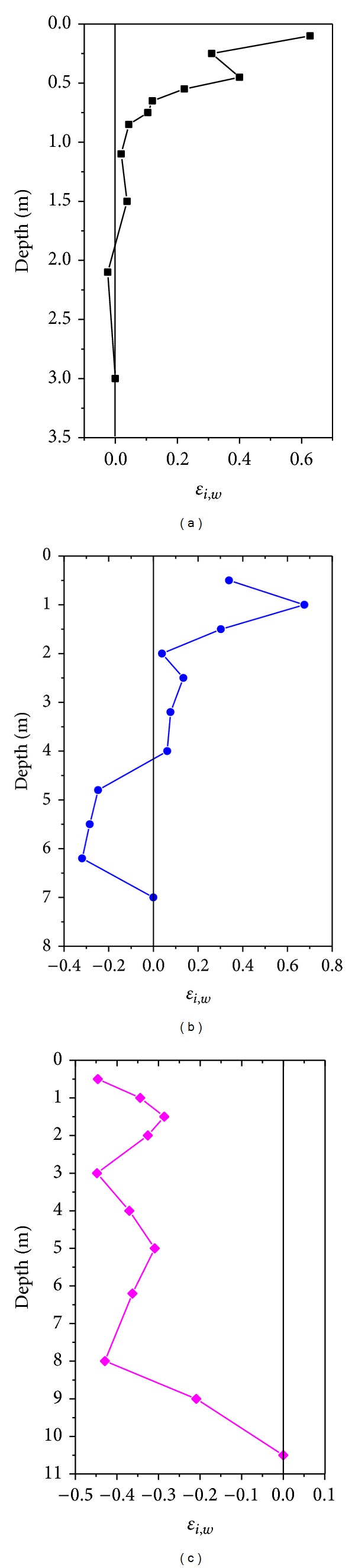
Calculated strains of black shale by immobile element Ti in Chengkou County. (a) Profile A, (b) profile B, and (c) profile C.

(2) εi,w=VwVp−1=ρpCi,pρwCi,w−1,

where *ρ* refers to bulk density. Other signs and subscripts are defined as in (1). Positive *ε*_*i*,*w*_ values indicate expansions in initial protolith volume, a value of zero represents iso-volumetric changes, and negative strains denote the collapse of weathered rocks. The *ε*_*i*,*w*_ versus depth for each profile is shown in Table 1 and Figure 4 from (2). At depths below 0.8 m in profile A, the value of *ε*_*i*,*w*_ is close to zero, thus indicating that weathering is essentially iso-volumetric in this situation. By contrast, *ε*_*i*,*w*_ values exceed zero at depths above 0.8 m, which suggests that the weathering samples are enriched at the regolith zone and at the upper half of the saprock zone. In profile B, the weathered samples collapsed at 4 m below the surface. However, *ε*_*i*,*w*_ value increases with decreasing depth, especially the maximum *ε*_*i*,*w*_ value can reach approximately 0.65, although this depth remains above 0.8 m in profile A and above 2 m in profile B. In profile C, *ε*_*i*,*w*_ values are less than zero, which indicates that the shale was compacted at all depths as they were established in geological weathering time.

### 4.5. Mass Profile Changes

The total mass flux of an element *j* that is added to or depleted from the weathered profile can be calculated by integrating the area under the curve of the *τ*_*i*,*j*_ plot. Elemental gain or loss *M*_*j*,flux_ (amount of substance length^−2^) is computed by integrating weathered rock depth *z* with *ε*_*i*,*w*_ and *τ*_*i*,*j*_ plots. The specific equation is as follows [21, 25–27]:

(3) Mj,flux=ρpΔzCj,pτj,wmj=ρpCj,pmj∫0Lτi,j(z)(εi,w(z)+1)dz.

Here, units for *C*_*j*,*p*_, *ρ*_*p*_, *m*_*j*_, and *z* are *M* *M* ^−1^, *M* *L* ^−3^, *M* *N* ^−1^, and *L* (where *L* is any length scale and *N* is amount of substance scale), respectively. In Equation (3), *m*_*j*_ is the atomic weight of species *j* and *L* is applied throughout the current full depth of weathered shale. We equated this depth to that of protolith. Although the results for certain weathered samples can be highly inaccurate if the changes in bulk density or strain are not incorporated into *M*_*j*,flux,_ and this correction is not commonly applied [25, 28].

The mass fluxes of major elements are listed in Table 4. The alkali and alkaline earth elements are almost completely depleted in all profiles. The mass flux of Ca reached 815 mol m^−2^ in the saprock zone of profile A because of the calcite and gypsum preserved in this material. Mg was depleted at all zones in all three profiles, thus indicating that Mg-bearing minerals were leached from the rock and transported by fluid-rock interaction in the form of Mg^2+^. Similarly, alkali elements were leached in all three profiles. Moreover, Na and K were depleted by up to −1322 and −238 mol m^−2^, respectively, along the entire transect (Table 4). Si was enriched by a maximum of 813 mol m^−2^ across this transect in profile A. However, the mass flux of Si was depleted in profiles B and C, which indicates that profile C has reached the Si removal stage. This profile also displayed the strongest degree of weathering among the three. Hence, the intense leaching of Si species by the hydrolysis of silicate minerals dominates chemical gains and losses. The calculated maximum mass fluxes of Al are 47, −65, and 207 mol m^−2^ in the regolith zones of profiles A, B, and C, respectively, as integrated over sampling depth. Furthermore, Al is accumulated in this zone because aluminous clay is partially filled with this oriented material. The maximum mass fluxes of Fe are −1041, −198, and −1786 mol m^−2^ in profiles A, B, and C, respectively, as integrated over sampling depth. This result suggests that Fe is locally redistributed from weathered shale to regolith. In shale evolution studies, the relative number of unstable and stable phases predicts future trends in mass flux. If the shale contains many weatherable minerals, elemental mass fluxes may remain high. In addition, collapses are influenced by the depletion of Si, Ca, and Mg through the dissolution of plagioclase, muscovite, calcite, and dolomite.

**Figure 5:**
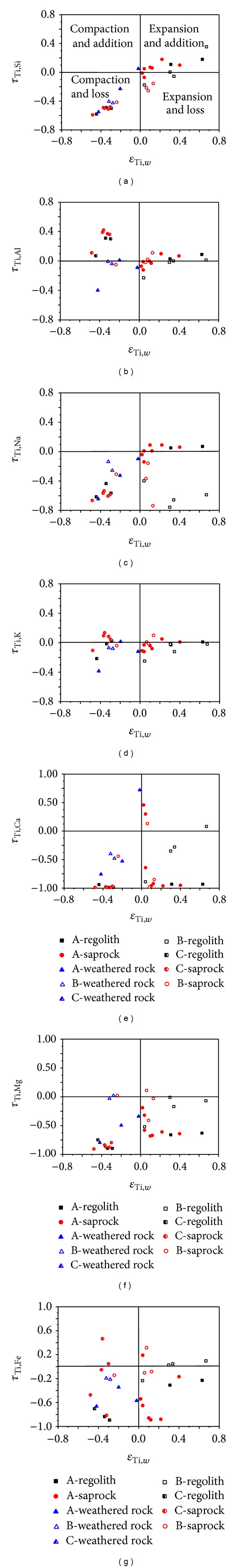
Mass transfer coefficient *τ* of (a) Si, (b) Al, (c) Na, (d) K, (e) Ca, (f) Mg, and (g) Fe versus strain calculated using Ti.

Following the computation of mass flux, we evaluate the strain versus*τ* plots for each element (Figure 5) because elements that potentially contribute to deformation are plotted with nonzero slopes. These plots can be divided into four quadrants: the upper left quadrant represents compaction as measured by negative *ε*_Ti,*w*_ values and the addition of a particular element as measured by positive *τ* values, the lower left quadrant indicates compaction and loss, the upper right quadrant denotes expansion and addition, and the lower right quadrant corresponds to the expansion and loss of the element under consideration. Si is enriched in the regolith zones of profiles A and B but is depleted at all depths in profile C (Figure 5(a)). It is reduced from saprock to regolith in profiles A and B but collapses at all depths in profile C. Al concentration increases in the regolith zones of all three profiles but decreases in the saprock zone of profile A (Figure 5(b)). Na is enriched by residue in the saprock and regolith zones of profile A. However, more than 50% of Na is leached at all depths in profiles B and C (Figure 5(c)). K is leached from weathered rock to regolith in all three profiles, with the exception of the saprock zone in profile C (Figure 5(d)). The alkaline earth elements are almost completely depleted from weathered rock to regolith in all three profiles (Figures 5(e) and 5(f)). Fe increases in part of the saprock zone because Fe oxyhydroxide or oxide is preserved in this area. In addition, Fe is enriched in the regolith zone of profile B (Figure 5(g)). This finding is attributed to the possibility that microorganisms or roots may have been involved in the interaction. We interpret the deformational history of and strain generation in the study area as follows. The expansion in profile A is ascribed to considerable additions of Si and some Al; the compaction in weathered rock and the bottom of the saprock zone in profile B and at all depths in profile C is attributed to the leaching of major elements such as Na, Ca, Mg, and Si. The expansion in the saprock and regolith zones of profile B is ascribed to considerable incorporation of Al and Fe, as well as biochemical effects.

### 4.6. Mechanism of Chemical Weathering

**Figure 6:**
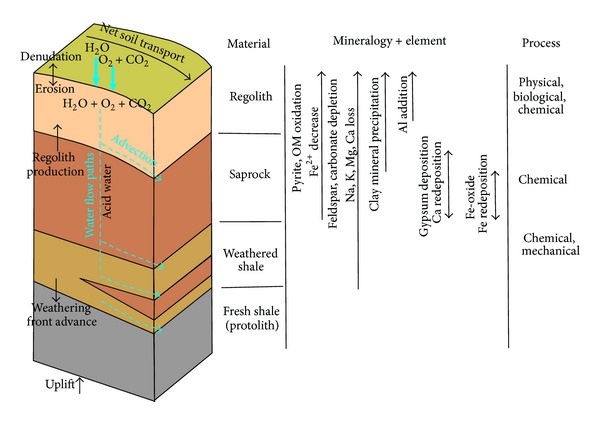
Conceptual weathering model of Shuijingtuo Formation black shale, Lower Cambrian.

The conceptual model of the weathering mechanism of black shale is illustrated in Figure 6. First, surface water infiltrates the regolith zone with O_2_ and CO_2_. Pyrite and organic matters are oxidized by dissolved oxygen to produce sulfate-type and organic-type acid water. In addition, CO_2_ dissolves in water to generate carbonate-type acid water. This acid water completely destroys shale texture, leaches alkali and alkaline earth elements, and produces the clay mineral in which Al accumulates in the regolith zone. In this zone, the surface soil is transported by either physical processes involving surface water or a slope gravitational process. The biological, physical, and chemical processes expose new avenues by which acid water can flow through the regolith zone. The regolith front moves downward, and regolith material is produced at this front [29, 30]. Second, acid water flows through the saprock zone to extract and transport chemical components such as alkali and alkaline earth elements. However, Ca and Fe are redeposited as gypsum and Fe-oxide, respectively, at the bottom half of the saprock zone. The water flows vertically through the saprock and in advection at the base of the saprock zone. The chemical processes corrode and disintegrate the shale to improve porosity and permeability. Third, acid water is gradually consumed in the saprock zone, and groundwater infiltrates the weathered shale zone. The minerals are hydrolyzed and hydrated to leach the silicate mineral. A certain saprock material is formed along the joints, bedding, and small cracks in the weathered shale zone because the groundwater permeates and interacts with the shale along the cracks. In this zone, mechanical and chemical processes are interrupted and interact with the shale along the margin of the cracks. As ions, the elements flow by the advection of aqueous media, and the weathering front is formed at the bottom of the weathered shale zone.

## 5. Conclusions

In this study, we analyzed the elemental transportation, elemental correlation, volumetric strain, mass fluxes, and weathering process of black shale from the Shuijingtuo Formation of the Lower Cambrian in Chengkou County, Chongqing Province. The following conclusions can be drawn from the discussion above.

1. Alkali and alkaline earth elements are leached from fresh shale, and the mass transportation pattern is classified under the “depletion” pattern. Fe is depleted at the weathered shale zone and increases at the saprock zone. The mass transportation pattern is considered a “depletion-addition” pattern. Al is accumulated in the regolith zone, which is accumulated in profile C. This accumulation can then be classified under the “addition” pattern.
2. In profiles A and B, expansion occurred in the regolith zone and in part of saprock zone, whereas compaction was observed in the weathered shale zone. Collapse occurred at all depths in profile C. As per PCA, elemental mobility and low pH enhance the weathering process. The mass fluxes are also related to degree of weathering. In chemical weathering, Na, K, Ca, Mg, and Si are depleted in the following sequence: valley (C) > near mountaintop (B) > ridge (A).

## Figures and Tables

**Table 1:** Major element concentration, bulk densities, strain values, and corresponding Tau values for black shale from profile A, B, and C in study area.

Sample No.	Depth (m)	*ρ* (g/cm^3^)	SiO_2_ (%)	Al_2_O_3_ (%)	TFe_2_O_3_ (%)	K_2_O (%)	Na_2_O (%)	CaO (%)	MgO (%)	MnO (%)	P_2_O_5_ (%)	BaO (%)	SO_3_ (%)	LOI (%)	Ti (ppm)	pH	ε_i,w_	τ_Ti,j_						
																		Si	Al	Fe	K	Na	Ca	Mg
A-1	0.1	1.53	65.5	13.60	8.10	2.84	2.52	0.29	0.97	0.22	0.20	0.53	0.23	4.39	4970	6.03	0.63	0.18	0.09	−0.23	0.01	0.07	−0.93	−0.63
A-2	0.25	1.79	65.7	13.70	7.78	2.87	2.63	0.28	0.96	0.05	0.25	0.47	0.30	4.41	5275	4.25	0.31	0.11	0.03	−0.31	−0.03	0.05	−0.93	−0.66
A-3	0.45	1.72	63.4	13.85	9.08	2.91	2.59	0.22	0.98	0.27	0.28	0.37	1.03	4.97	5138	3.28	0.40	0.10	0.07	−0.17	0.01	0.06	−0.95	−0.64
A-4	0.55	1.92	69.8	14.60	1.35	3.13	2.74	0.19	1.09	0.01	0.03	0.28	1.10	5.69	5271	2.82	0.22	0.18	0.10	−0.88	0.05	0.09	−0.96	−0.61
A-5	0.65	1.90	69.1	14.25	1.38	3.02	2.79	0.36	1.02	BDL	0.02	0.26	1.78	7.33	5818	2.68	0.12	0.06	−0.03	−0.89	−0.08	0.01	−0.92	−0.67
A-6	0.75	1.98	67.8	14.20	1.63	3.05	2.94	0.19	0.96	BDL	0.02	0.29	3.36	8.21	5657	3.40	0.10	0.07	0.00	−0.86	−0.04	0.09	−0.96	−0.68
A-7	0.85	2.22	62.6	13.35	3.97	2.93	2.56	1.58	1.20	0.02	0.09	0.46	7.26	8.56	5340	4.24	0.04	0.05	−0.01	−0.65	−0.03	0.01	−0.64	−0.58
A-8	1.1	2.42	55.7	11.70	4.93	2.51	2.28	5.94	2.16	0.05	0.17	0.32	8.31	10.64	5017	5.88	0.02	−0.01	−0.07	−0.54	−0.11	−0.04	0.46	−0.19
A-9	1.5	2.50	49.6	10.60	12.06	2.36	1.94	5.05	1.72	0.04	0.14	0.28	8.70	11.06	4767	6.12	0.04	−0.07	−0.12	0.19	−0.12	−0.14	0.30	−0.32
A-10	2.1	2.61	57.2	11.10	4.45	2.41	2.08	6.79	1.72	0.05	0.15	0.37	7.75	10.94	4859	6.88	−0.02	0.05	−0.09	−0.57	−0.12	−0.10	0.72	−0.34
A-11	3.0	2.65	52.3	11.75	9.93	2.63	2.22	3.80	2.49	0.05	0.18	0.33	6.25	10.76	4670	6.52	0.00	0.00	0.00	0.00	0.00	0.00	0.00	0.00

B-1	0.5	1.67	69.7	9.85	3.92	2.06	0.66	3.48	1.20	0.02	1.34	1.02	0.40	5.02	3542	7.04	0.34	−0.06	0.00	0.04	−0.13	−0.66	−0.28	−0.17
B-2	1.0	1.90	69.9	6.98	2.87	1.62	0.56	3.65	0.94	0.01	0.60	0.65	0.26	8.80	2486	6.55	0.67	0.35	0.01	0.09	−0.03	−0.59	0.08	−0.07
B-3	1.5	1.88	67.4	8.76	3.49	2.09	0.42	2.85	1.30	0.02	0.65	1.60	0.70	11.70	3234	6.62	0.30	0.00	−0.02	0.02	−0.03	−0.76	−0.35	−0.01
B-4	2.0	1.75	74.7	9.34	3.50	2.16	1.41	0.66	0.86	BDL	0.15	0.48	0.33	5.91	4355	6.20	0.04	−0.18	−0.23	−0.24	−0.26	−0.40	−0.89	−0.52
B-5	2.5	1.82	67.2	11.75	3.72	2.80	0.55	0.77	1.52	0.01	0.17	1.66	0.86	8.09	3834	6.02	0.13	−0.16	0.11	−0.09	0.09	−0.74	−0.85	−0.03
B-6	3.2	1.72	66.0	12.10	5.94	2.80	1.96	0.12	1.03	0.01	0.12	0.41	8.24	8.88	4275	4.43	0.08	−0.26	0.02	0.31	−0.02	−0.16	−0.98	−0.41
B-7	4.0	1.98	60.8	10.25	3.55	2.51	1.29	5.76	1.70	0.03	0.17	0.95	5.93	10.11	3764	6.50	0.06	−0.22	−0.02	−0.11	0.00	−0.37	0.13	0.11
B-8	4.8	2.14	59.7	13.00	4.42	3.11	1.84	3.74	2.04	0.03	0.20	0.52	7.00	9.01	4916	6.38	−0.24	−0.42	−0.05	−0.15	−0.05	−0.31	−0.44	0.02
B-9	5.5	2.21	59.4	13.35	4.13	3.06	2.01	3.56	2.09	0.03	0.20	0.46	6.47	9.01	5010	6.51	−0.28	−0.43	−0.04	−0.22	−0.09	−0.26	−0.48	0.02
B-10	6.2	2.38	59.7	13.35	4.13	3.01	2.29	4.00	1.94	0.04	0.19	0.50	5.83	8.37	4886	6.80	−0.32	−0.41	−0.01	−0.20	−0.08	−0.14	−0.40	−0.03
B-11	7.0	2.50	66.0	8.77	3.36	2.12	1.72	4.30	1.29	0.02	0.17	0.37	5.34	9.30	3167	7.12	0.00	0.00	0.00	0.00	0.00	0.00	0.00	0.00

C-1	0.5	1.56	65.5	15.30	2.73	2.87	0.23	2.73	2.03	0.22	0.79	0.20	0.18	8.99	6345	6.54	−0.44	−0.58	0.07	−0.71	−0.22	−0.62	−0.94	−0.75
C-2	1.0	1.49	70.5	16.60	1.33	3.19	0.30	0.31	0.74	0.02	0.04	0.83	0.30	5.01	5611	6.40	−0.34	−0.49	0.31	−0.84	−0.02	−0.44	−0.99	−0.90
C-3	1.5	1.35	69.8	16.70	0.87	3.38	0.23	0.41	0.77	0.01	0.03	0.59	0.18	5.84	5693	6.16	−0.29	−0.50	0.30	−0.90	0.02	−0.57	−0.99	−0.90
C-4	2.0	1.43	68.7	17.50	1.48	3.55	0.21	0.31	0.86	0.01	0.04	0.55	0.16	5.42	5689	6.35	−0.32	−0.51	0.37	−0.82	0.08	−0.61	−0.99	−0.88
C-5	3.0	1.57	63.9	15.85	4.82	3.28	0.20	0.37	0.77	0.01	0.04	0.61	4.16	9.26	6329	3.85	−0.48	−0.59	0.11	−0.48	−0.11	−0.67	−0.99	−0.91
C-6	4.0	1.79	60.5	15.20	6.73	3.08	0.20	0.63	1.02	0.14	0.14	0.63	5.25	10.60	4867	3.70	−0.37	−0.49	0.39	−0.06	0.09	−0.57	−0.98	−0.84
C-7	5.0	1.71	58.8	14.20	7.09	2.81	0.18	0.94	1.18	0.30	0.10	0.79	6.00	10.45	4643	4.74	−0.30	−0.48	0.36	0.04	0.04	−0.59	−0.97	−0.80
C-8	6.2	1.86	56.9	14.85	9.91	3.02	0.20	0.35	0.78	0.03	0.08	0.46	6.10	11.61	4627	4.65	−0.36	−0.50	0.42	0.46	0.13	−0.54	−0.99	−0.87
C-9	8.0	1.90	55.3	6.87	2.46	1.79	0.17	6.80	1.30	0.02	3.90	0.46	4.07	13.89	5055	6.83	−0.42	−0.55	−0.40	−0.67	−0.39	−0.65	−0.76	−0.80
C-10	9.0	2.45	53.6	6.45	2.71	1.66	0.18	8.00	1.84	0.02	1.74	0.35	4.18	17.53	2831	7.22	−0.20	−0.23	0.01	−0.35	0.01	−0.33	−0.53	−0.50
C-11	10.5	2.74	49.1	4.51	2.94	1.16	0.19	13.5	2.60	0.01	2.90	0.35	2.77	19.32	2002	7.26	0.00	0.00	0.00	0.00	0.00	0.00	0.00	0.00
UCC			66.0	15.20	5.00	3.40	3.90	4.20	2.20	0.06	0.50													

Note: BDL, below detection limit: <0.01%.

**Table 2:** Pearson correlation coefficients among major elements, density, and pH value for the profiles A, B, and C, respectively. Values greater than 0.80 are indicated in bold.

	SiO_2_	Al_2_O_3_	Fe_2_O_3_	K_2_O	Na_2_O	CaO	MgO	P_2_O_5_	SO_3_	LOI	*ρ*
Profile A											
Al_2_O_3_	**0.955** ∗∗										
Fe_2_O_3_	−0.683∗	−0.556									
K_2_O	**0.916** ∗∗	**0.983** ∗∗	−0.583								
Na_2_O	**0.930** ∗∗	**0.964** ∗∗	−0.651∗	**0.958** ∗∗							
CaO	**−0.843** ∗∗	**−0.939** ∗∗	0.275	**−0.925** ∗∗	**−0.877** ∗∗						
MgO	**−0.852** ∗∗	**−0.806** ∗∗	0.365	−0.745∗∗	−0.749∗∗	**0.822** ∗∗				.	
P_2_O_5_	−0.359	−0.286	0.760∗∗	−0.387	−0.394	0.141	0.160				
SO_3_	**−0.812** ∗∗	**−0.842** ∗∗	0.181	−0.762∗∗	−0.737∗∗	**0.874** ∗∗	0.754∗∗	−0.093			
LOI	−0.780∗∗	**−0.815** ∗∗	0.102	−0.725∗	−0.678∗	**0.863** ∗∗	**0.813** ∗∗	−0.219	**0.944** ∗∗		
*ρ*	**−0.814** ∗∗	**−0.836** ∗∗	0.180	−0.741∗∗	−0.740∗∗	**0.877** ∗∗	**0.869** ∗∗	−0.093	**0.914** ∗∗	**0.955** ∗∗	
pH	**−0.809** ∗∗	**−0.868** ∗∗	−0.569	**−0.879** ∗∗	**−0.864** ∗∗	0.794∗∗	0.725∗	0.414	0.622∗	0.588	0.626∗

Profile B											
Al_2_O_3_	0.710∗										
Fe_2_O_3_	−0.312	0.665∗									
K_2_O	−0.754∗∗	**0.980** ∗∗	0.631∗								
Na_2_O	−0.593	0.674∗	0.548	0.672∗							
CaO	−0.543	−0.097	−0.434	−0.074	0.098						
MgO	**−0.917** ∗∗	−0.758∗∗	0.146	0.786∗∗	0.484	0.487					
P_2_O_5_	0.373	−0.414	−0.238	−0.546	−0.605∗	0.159	−0.301				
SO_3_	−0.781∗∗	0.663∗	0.665∗	0.711∗	**0.847** ∗∗	0.218	0.563	−0.573			
LOI	−0.478	−0.021	−0.031	0.118	0.021	0.312	0.300	−0.365	0.346		
*ρ*	−0.626∗	0.264	−0.157	0.314	0.595	0.575	0.590	−0.426	0.509	0.367	
pH	−0.074	−0.291	−0.764∗∗	−0.320	−0.198	0.712∗	0.254	0.398	−0.344	−0.061	0.473

Profile C											
Al_2_O_3_	**0.873** ∗∗										
Fe_2_O_3_	−0.376	0.107									
K_2_O	**0.867** ∗∗	**0.990** ∗∗	0.102								
Na_2_O	0.740∗∗	0.561	−0.383	0.501							
CaO	−0.781∗∗	**−0.949** ∗∗	−0.249	**−0.962** ∗∗	−0.422						
MgO	−0.639∗	−0.745∗∗	−0.190	**−0.810** ∗∗	−0.321	**0.866** ∗∗					
P_2_O_5_	−0.691∗	**−0.901** ∗∗	−0.271	**−0.884** ∗∗	−0.477	**0.855** ∗∗	0.641∗				
SO_3_	−0.662∗	−0.299	**0.840** ∗∗	−0.266	−0.668∗	0.072	−0.044	0.110			
LOI	**−0.968** ∗∗	**−0.932** ∗∗	0.194	**−0.930** ∗∗	−0.670∗	**0.875** ∗∗	0.750∗∗	0.736∗∗	0.513		
*ρ*	**−0.913** ∗∗	**−0.907** ∗∗	0.108	**−0.916** ∗∗	−0.510	**0.901** ∗∗	0.766∗∗	0.671∗	0.392	**0.963** ∗∗	
pH	−0.159	−0.548	−0.728∗	−0.569	0.099	0.648∗	0.581	0.609∗	−0.552	0.300	0.381

Data from the three profiles. **P < 0.01, *P < 0.05.

**Table 3:** Correlations among major elements, density, and pH value with principal components.

	Profile A				Profile B				Profile C			
	PC1	PC2	PC3	PC4	PC1	PC2	PC3	PC4	PC1	PC2	PC3	PC4
SiO_2_	−0.967	0.137	−0.193	0.077	−0.892	−0.345	−0.050	−0.265	−0.921	0.378	−0.042	−0.037
Al_2_O_3_	−0.989	0.051	0.080	0.101	0.862	−0.253	0.384	0.079	−0.983	−0.078	0.091	−0.126
Fe_2_O_3_	0.525	−0.771	0.282	−0.195	0.569	−0.716	0.098	0.145	−0.003	−0.942	0.227	0.026
K_2_O	−0.955	0.160	0.193	0.082	0.906	−0.223	0.232	0.102	−0.984	−0.108	−0.014	−0.105
Na_2_O	−0.946	0.206	0.095	0.119	0.833	−0.095	0.015	−0.470	−0.618	0.529	0.439	0.367
CaO	0.945	0.171	−0.220	0.068	0.198	0.893	0.047	0.102	0.952	0.263	0.069	−0.010
MgO	0.878	0.128	0.238	0.371	0.806	0.403	0.280	0.222	0.813	0.283	0.322	−0.349
P_2_O_5_	0.265	−0.899	−0.010	0.203	−0.629	0.177	0.533	0.340	0.857	0.240	−0.346	0.144
SO_3_	0.872	0.385	0.040	−0.140	0.907	−0.083	−0.163	−0.042	0.345	−0.914	−0.064	0.152
LOI	0.852	0.495	0.100	−0.041	0.332	0.315	−0.731	0.472	0.968	−0.203	0.091	−0.001
*ρ*	0.883	0.397	0.146	0.068	0.588	0.635	−0.165	−0.382	0.941	−0.086	0.252	0.065
pH	0.864	−0.242	−0.266	0.139	−0.216	0.899	0.306	−0.156	0.490	0.808	−0.051	0.043
Eigen value	8.741	2.146	0.378	0.303	5.787	3.062	1.260	0.899	7.651	3.073	0.560	0.335
Variance explained (%)	72.840	17.882	3.154	2.524	48.222	25.518	10.503	7.489	63.758	25.605	4.670	2.795
Cumulative variance (%)	72.840	90.721	93.875	96.400	48.222	73.740	84.243	91.731	63.758	89.363	94.033	96.828

**Table 4:** Mass fluxes (mol/m^2^) of major elements in three profiles.

Materials	Depth (m)	Si	Al	Fe	K	Na	Ca	Mg
Profile A								
Regolith	0~0.25	285	47	−45	0	7	−191	−100
Saprock	0.25~1.5	334	−57	−563	−44	−10	−662	−476
Weathered rock	1.5~3.0	194	−167	−433	−78	−82	815	−349
	0~3.0	813	−177	−1041	−122	−85	−38	−925
Profile B								
Regolith	0~2.0	−723	−65	−1	−84	−506	−762	−129
Saprock	2.0~4.8	−8750	−19	−17	−22	−536	−1984	−155
Weathered rock	4.8~7.0	−13312	−91	−180	−90	−280	−1483	−1
	0~7.0	−22785	−175	−198	−196	−1322	−4229	−285
Profile C								
Regolith	0~1.5	−10156	207	−674	−119	−94	−11479	−2141
Saprock	1.5~6.2	−29304	1499	−615	66	−280	−34617	−6840
Weathered rock	6.2~10.5	−19906	−173	−497	−185	−174	−19043	−4169
	0~10.5	−59366	1533	−1786	−238	−548	−65139	−13150
